# Ribosomal trafficking is reduced in Schwann cells following induction of myelination

**DOI:** 10.3389/fncel.2015.00306

**Published:** 2015-08-19

**Authors:** James M. Love, Sameer B. Shah

**Affiliations:** ^1^Fischell Department of Bioengineering, University of MarylandCollege Park, MD, USA; ^2^Departments of Orthopaedic Surgery and Bioengineering, University of California, San DiegoLa Jolla, CA, USA

**Keywords:** ribosome, transport, Schwann cell, neuron, myelination, live imaging, modeling

## Abstract

Local synthesis of proteins within the Schwann cell periphery is extremely important for efficient process extension and myelination, when cells undergo dramatic changes in polarity and geometry. Still, it is unclear how ribosomal distributions are developed and maintained within Schwann cell projections to sustain local translation. In this multi-disciplinary study, we expressed a plasmid encoding a fluorescently labeled ribosomal subunit (L4-GFP) in cultured primary rat Schwann cells. This enabled the generation of high-resolution, quantitative data on ribosomal distributions and trafficking dynamics within Schwann cells during early stages of myelination, induced by ascorbic acid treatment. Ribosomes were distributed throughout Schwann cell projections, with ~2-3 bright clusters along each projection. Clusters emerged within 1 day of culture and were maintained throughout early stages of myelination. Three days after induction of myelination, net ribosomal movement remained anterograde (directed away from the Schwann cell body), but ribosomal velocity decreased to about half the levels of the untreated group. Statistical and modeling analysis provided additional insight into key factors underlying ribosomal trafficking. Multiple regression analysis indicated that net transport at early time points was dependent on anterograde velocity, but shifted to dependence on anterograde duration at later time points. A simple, data-driven rate kinetics model suggested that the observed decrease in net ribosomal movement was primarily dictated by an increased conversion of anterograde particles to stationary particles, rather than changes in other directional parameters. These results reveal the strength of a combined experimental and theoretical approach in examining protein localization and transport, and provide evidence of an early establishment of ribosomal populations within Schwann cell projections with a reduction in trafficking following initiation of myelination.

## Introduction

Schwann cells (SCs), a major cell type of the peripheral nervous system, are tasked with supporting neurons in numerous ways, from enhancing electrical conduction (Waxman and Bennett, 1972) to providing trophic factors for axonal growth and regeneration (Son and Thompson, 1995; Bryan et al., 2000). In this capacity, they possess a distinctive bipolar architecture; internode distances of up to 500 μm require distances between the Schwann cell body and distal processes to reach over 200 μm (Jacobs and Cavanagh, 1969). As in neurons, another polarized cell of unusual geometry (Twiss and van Minnen, 2006), myelinating and unmyelinating SCs appear to synthesize particular proteins at the site of demand within projections, rather than in the cell body (Gould and Mattingly, 1990).

In contrast to ER-bound ribosomes, which are mainly tasked with the production of membrane-associated or secreted proteins (Caro and Palade, 1964; Jamieson and Palade, 1967; Leader, 1979), cytosolic protein synthesis occurs on free ribosomes and is typically responsible for producing many proteins locally, including intracellular signaling molecules, transcription factors, and cytoskeletal elements (Ganoza and Williams, 1969). Evidence of local protein synthesis in myelinating cells remains mostly indirect, based on the presence of mRNA in isolated polysomes and granules within both unmyelinating and myelinating glial cells (Brophy et al., 1993). Additionally, myelin basic protein (MBP) mRNA has been observed to be distributed throughout SC and oligodendrocyte projections (Trapp et al., 1987, 1988; Griffiths et al., 1989; Gould and Mattingly, 1990), and radiolabeled MBP (2 min) appears early compared to the cell body-synthesized protein PLP (30 min) in the myelin fraction of oligodendrocytes, providing additional evidence for translation of MBP within the myelin fraction (Colman et al., 1982).

Lacking from our current understanding is how these free ribosomal populations are distributed in Schwann cells during development and how ribosomal distribution is influenced by Schwann cell interactions with neurons. Developing a better understanding of these mechanisms may provide better insight into protein synthetic dynamics as well as the stability of observed ribosomal structures, in preparation for translation of Schwann cell proteins as well as, intriguingly, those potentially transmitted to neurons (Court et al., 2008, 2011). We used quantitative biological and theoretical approaches to probe the hypothesis that the induction of myelination would increase trafficking of ribosomes, to account for the required increase in production of myelin associated proteins. Expression of fluorescently labeled ribosomal subunits in primary Schwann cells allowed us to track ribosomal transport in real time, and quantify changes in patterns of ribosomal localization and transport during both their initial interactions with neurons in culture and in early phases of myelination. Multiple regression analysis and the development of a data-driven rate kinetic model enabled us to develop further insight into these changes. Our data suggest that stable ribosome-enriched foci appear early during Schwann cell projection extension and are maintained during early stages of myelination. However, transport of new ribosomes into projections slows upon induction of myelination, primarily due to increased conversion of anterograde (outward) moving particles to a stationary pool.

## Materials and methods

### Animal usage and euthanasia

Animal usage was in accordance with protocols approved by the UCSD Institutional Animal Care and Use Committee (IACUC). Pregnant female rats were euthanized by asphyxiation with CO_2_ followed by confirmation via secondary means (removal of major organs). P2 neonates were incapacitated with CO_2_ followed by euthanization via decapitation.

### DRG collection and culture

**Figure 1:**
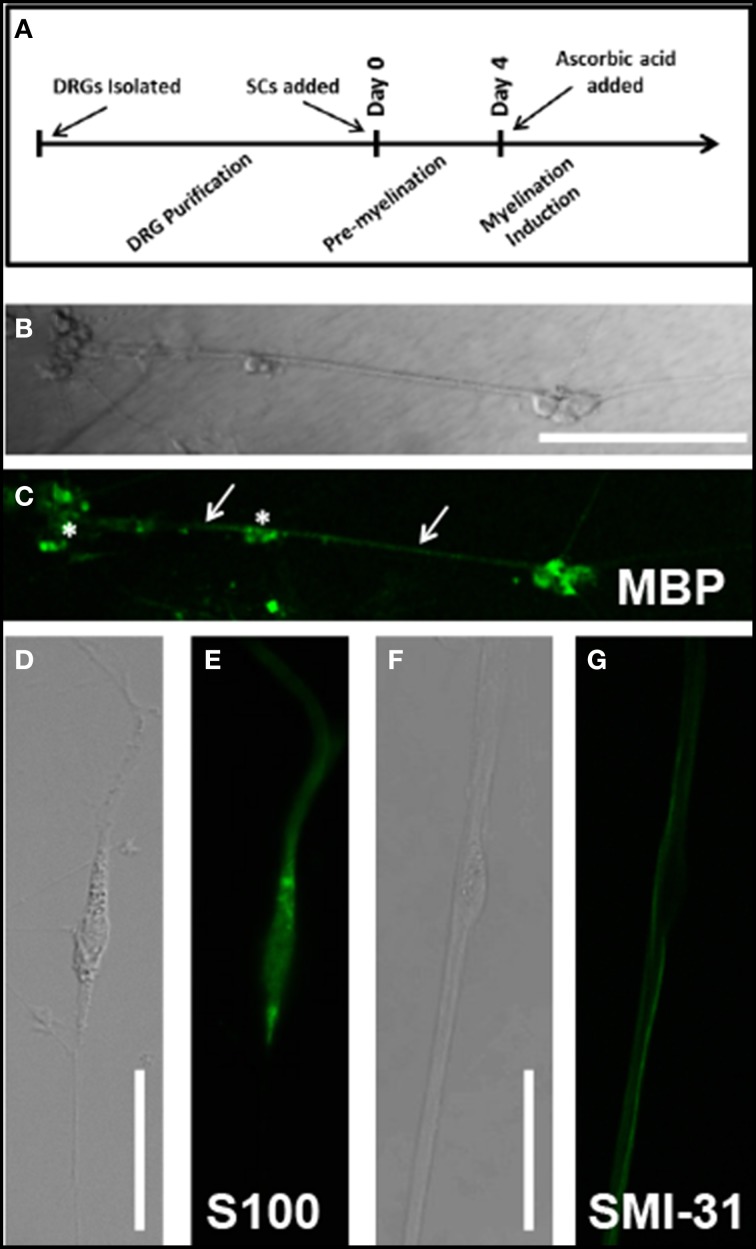
**(A)** Cell culture timeline. All experimental days reference days since Schwann cell addition to neuronal culture; Confirmation of myelination protocols and cell culture techniques was observed through immunolabeling: **(B)** DIC image of Day 14AA culture indicates several clusters of Schwann cells and neurons (stars) as well as fascicles of myelinated DRG axons (arrows); **(C)** MBP immunostain of Day 14AA culture shows low levels of MBP expression, indicative of early myelination; **(D)** DIC image of cultured Schwann cells at Day 1 **(E)** S100 immunostain identifies cultured Schwann cells; **(F)** DIC image of a single Schwann cell (star) abutting neuronal processes; **(G)** SMI-31 (phosphorylated neurofilament) immunostain identifies location of neuronal processes relative to Schwann cell body (star); Scale bar B/C: 200 μm, D/E/F/G: 50 μm.

DRG culture methods were adapted from previously published methods (Lee et al., 1995). Dorsal root ganglia (DRGs) were removed from E15 Sprague-Dawley rats in HBSS. DRGs were dissociated in trypsin for 30 min at 37°C. Following trypsinization, samples were washed 3x in fresh pre-warmed media. DRGs (~30,000 per plate) were plated on laminin-coated glass coverslips placed within 24-well plates supplemented with 500 μl of prewarmed DRG media consisting of MEM with 10% FBS, 2% B27, 1 ng/ml NGF, and 1% Penicillin/Streptomycin. One day post-plating, culture media was supplemented with 1 ug/ml FdU to eliminate contaminating fibroblasts and Schwann cells. Media was alternated every 2–3 days between FdU application and fresh media for a period of 2 weeks to obtain a mostly purified neuronal culture (Figure 1A).

### Schwann cell isolation

Schwann cells were isolated and cultured per previously published methods (Lee et al., 1995). Primary Schwann cells were obtained from the sciatic nerves of ~10 P2 Sprague-Dawley rat pups (20 nerves). Nerves were dissected into 14 ml of L15 media then spun down at 3000 rpm for 2 min. Media was aspirated off and nerves were resuspended in 2 ml L-15 and 1 ml 10 mg/ml collagenase type P and incubated at 37°C for 45 min, mixing every 10 min. Tissue was spun down for 2 min at 1000 × g, media aspirated off and pre-warmed digestion solution added (3 ml 0.25% trypsin and 1 ml DNase [1.2 mg/ml]) Sample was incubated for 15 min at 37°C. Following incubation, the reaction was halted by supplementing sample with 5 mL DMEM and 10% FBS. Sample was centrifuged for 2 min at 1000 × g followed by two washes with 10 ml of ice cold L15 media followed by 1000 × g centrifugations. Samples were supplemented with 5 ml of media and triturated. Cells were spun down at 1000 × g and media removed for transfection.

### Transfection

Nucleofection was performed according to manufacturer's instructions (4D-Nucleofector™ System, Lonza AG, Switzerland) using P3 primary cell solution and program setting DC-100 to transfect. Either 3 μg of L4-GFP plasmid (a generous gift of Dr. Tim Krüger (Krüger et al., 2007) or control GFP plasmid (Lonza AG, Switzerland) were used.

Expression persisted for up to 2 weeks in culture. However, the percentage of cells expressing the plasmid decreased over time, as has been previously reported (Pedraza and Colman, 2000). Thus, our experimental time course was conservatively chosen to span early phases of myelination, and includes cells from 1 day post-transfection (Day 1) to cells 1 week post-transfection (Day 7) (Figure 1A).

### Induction of myelination

Myelination was induced with ascorbic acid per published protocols (Eldridge et al., 1987). On the fourth day of co-culture with neurons, Schwann cell media was supplemented with 50 μg/ml ascorbic acid to initiate the process of myelination. Cells treated with ascorbic acid and observed were designated (Day 7AA) in contrast with untreated cells (Day 7 No AA). Myelination was confirmed using immunocytochemistry at day 14 (see Figures 1B,C). Schwann cells in culture were identifiable by immunostaining against S100 (see Figures 1D,E) while neurons were identified by immunostaining against SMI-31 phosphorylated neurofilament (see Figures 1F,G).

### Immunocytochemistry

Immunocytochemistry was performed at various developmental time points, using published protocols (Love et al., 2012). Samples were labeled with one or a combination of the following primary antibodies: mouse anti-S100 (Sigma-Aldrich S2532), mouse anti-SMI31 (abcam ab24573), mouse anti-MBP, human anti-Ribo-P (Immunovision HPO-0100), mouse anti-RPL4 (Sigma-Aldrich WH0006124M1), mouse anti-tubulin (Sigma-Aldrich T9026). Appropriate species specific fluorescently-labeled secondary antibodies were used to visualize primary antibody localization. Secondary antibodies used included: Alexa Fluor 488-goat anti-mouse (Invitrogen A-11001), Alexa Fluor 594-goat anti-mouse (Invitrogen A-11032), Alexa Fluor 594-goat anti-rabbit (Invitrogen A-11037), and Alexa Fluor 594-goat anti-human (Jackson Immunoresearch 109-585-003). Desired samples were incubated for 30 min prior to mounting in Alexa Fluor 594-phalloidin (Invitrogen A-12381) to visualize actin.

Analysis for co-localization was performed using ImageJ plugin JACoP (Bolte and Cordelières, 2006). Images were analyzed for overlapping expression using the Manders coefficient, which incorporates both position and fluorescence intensity into assessment of co-localization (Manders et al., 1992).

### Imaging

Cells were imaged using a Leica SP5 system within an environmental chamber (Tokai Hit, Japan) that, for live-imaging experiments, enabled maintenance of the environment at 37°C and 5% CO_2_. Standard lasers and filters were used. An argon laser enabled excitation at 488 nm with HeNe lasers for 594 nm. Emission was captured between 500 and 550 nm (GFP, Alexa-Fluor 488) and 600–650 (Alexa-Fluor 594). Laser power and gains were adjusted to provide best images for each sample. Images were obtained using a 63x glycerol (80% glycerol/20% water) immersion objective with a pinhole of 1 airy (102.9 μm). Time-lapse images were taken at a resolution of 512 × 512 pixels with 3x line averaging. Stand-alone images were captured with a resolution of 1024 × 1024 pixels with 3x line averaging.

Time course images of transfected cells were captured at 5 s intervals for a period of 5 min. The capture rate and imaging period were chosen based upon several factors including anticipated transport rates, desired resolution, and minimization of photobleaching and phototoxicity.

### Image processing and analysis

Custom Matlab (Mathworks, Inc.) programs were modified from previous studies (Chetta and Shah, 2011; Love et al., 2012) to convert time course images into a kymograph. Kymographs allow for visualization of changes in an intensity profile of a 1-dimensional trace over time with the time variable displayed along the y-axis and cellular position along the x-axis. Kymograph traces in our analysis were made along the long axis of the SC projection allowing visualization of transport throughout the projection with a thickness of three pixels. The trace was suppressed to a single pixel thickness by choosing the maximum intensity value of the three. Kymographs were analyzed for both ribosomal distributions along the projection as well as transport characteristics of mobile ribosomal populations.

For characterization of ribosomal distributions, kymographs were averaged along the y-axis providing an average projection profile for the 5 min imaging duration. The profile was manually segmented to separate the cell body from the projection for characterization. Stable populations of ribosomes were noted within the projections through identification of peaks in the average intensity above background fluorescence levels (>3x). Peaks within 5 pixels (~2.5 μm) were counted as a single peak to minimize double counting. Average fluorescence levels were identified for both the cell body and projection regions. Peak distance values were determined from identified peak locations and specified projection start.

Transport parameters were computed based upon manual identification of mobile ribosomal particles with a continuous trajectory from frame to frame and an intensity of at least 3x above background. In addition to direction of movement in previous frames, intensity and line-width of a trajectory were also factored into particle tracking. Additionally, kymographs were blinded for experimental group. We used a conservative inclusion criteria in analyzing traces that potentially merged or split. Trajectories that appeared to merge were not double-analyzed in areas of potential overlap (i.e., one of the two trajectories was truncated at the merge site (or initiated at the split site). Thus, tracks were not incorrectly lengthened, but may have been prematurely truncated. Such track shortening was assumed to have minimal impact on results and their interpretation due to the following rationale: (i) average velocities, which are measured from directional runs would be unaffected; (ii) net velocities, which are weighted by track duration (shorter tracks contribute less) would be minimally affected; (iii) increases in directional populations for a given direction would inversely correlate with run duration (e.g., an increase in anterograde particles would correspond with a decrease in anterograde run duration). This was not observed. In no case did a significant increase or decrease in particle number correspond to a corresponding reduction or increase, respectively, in run duration. Mean particle parameters were computed and presented as average values. Net velocity was computed based on the aggregate distance traveled by all particles in a given sample divided by the time window of observation (Pathak et al., 2013). This provides a reasonable surrogate for the collective level of transport conducted by a cell throughout the duration of the 5-min imaging window.

### Multiple regression analysis

Multiple regression analysis was performed on the z-scored data of all individual traces within an experimental group. The dependent variable (net velocity) was regressed against the independent variables average velocity, total duration, anterograde velocity, anterograde duration, stationary velocity, stationary duration, retrograde velocity, and retrograde duration. Higher β-weights indicate a stronger contribution to the regression model.

### Modeling

A ribosomal transport model was implemented using a rate kinetics model to evaluate transitions from stationary, retrograde, and anterograde ribosomal populations. The model, modified slightly from its original form (Zadeh and Shah, 2010), used a system of coupled differential equations for each population:

(1) $$\frac{d[Ant]}{dt}=k_{+1}[Stat]-k_{-1}[Ant]$$

(2) $$\frac{d[Ret]}{dt}=k_{+2}[Stat]-k_{-2}[Ret]$$

The stationary population was set as an infinite source and sink with a value of 1 particle (Table 1). The model schematic can be seen in **Figure 7**. Particle quantities while modeled as concentrations within the equations refer to instantaneous particle counts. Initial values of anterograde and retrograde particles were calculated by dividing the average number of particles per experiment by the experimental duration (Table 1). The model was implemented in MATLAB (MathWorks, Natick, MA) using the differential equation solver ode45 which implements a Runge-Kutta method with a variable step size. In order to find a unique best solution, rates were varied between 0.001 and 1.001 in 0.02 steps for each rate constant (Table 2). Sensitivity analysis for each model parameter was performed by varying each parameter over its allowed range, and plotting model outputs. Best fit was determined by minimizing the mean square error (MSE) between modeled parameters and experimental data for net velocity and the number of particles in each state (Table 3). A maximum MSE of 0.02 for the net velocity fit was chosen such that the model fit within the range of standard errors observed experimentally. Initial model fit was made using the time points untreated with ascorbic acid (Days 1, 3, and 7). A fit was then made between the Day 4 time point of the untreated fit (time point at which ascorbic acid was added) and the Day 7 treated group. Within the model, net velocity was calculated by multiplying the instantaneous particle populations of each state by the instantaneous velocity of the population for a given time step and summing the two populations. Due to the lack of significant difference between anterograde and retrograde velocities, the average velocities across the four experimental time points was used as a constant throughout the model (Table 2). A velocity scaling factor (Table 2) was applied to allow for the starting net velocity of the model to match the experimental.

**Table 1:** **Model initial particle counts**.

	**Initial values**
Anterograde particles	0.4034
Retrograde particles	0.0782
Stationary particles	1

*Initial particle counts used to seed the model simulation*.

**Table 2:** **Model constants and rate variations**.

	**Model parameters**
Anterograde velocity (μm/s)	0.650782634
Retrograde velocity (μm/s)	−0.439211217
Velocity scaling factor	0.925432705
Minimum tested K-value (K_+1_, K_−1_, K_+2_, K_−2_)	0.001
Maximum tested K-value (K_+1_, K_−1_, K_+2_, K_−2_)	1.001
K-value iteration	0.02

*Directional velocities used to determine net velocities within the model, as well as, range over which rate constants were iterated during model fitting*.

**Table 3:** **Model results**.

	**Data**	**Model**	**Percent error**
**NET VELOCITY**			
Day 3	0.1981	0.2308	16.51
Day 7—No AA	0.7285	0.3027	10.50
Day 7—AA	0.1292	0.1314	1.70
**ANTEROGRADE PARTICLES**			
Day 3	0.3509	0.4671	33.11
Day 7—No AA	0.7285	0.5868	19.45
Day 7—AA	0.5096	0.4682	8.12
**RETROGRADE PARTICLES**			
Day 3	0.0808	0.1670	106.68
Day 7—No AA	0.3642	0.1806	50.41
Day 7—AA	0.3796	0.3949	4.03

*Model outputs and percent error for net velocity, anterograde particle count, and retrograde particle count*.

### Statistical analysis

In all cases a One-Way ANOVA was performed across all experimental groups (Day 1, Day 3, Day 7 No AA, Day 7 AA). Additionally, Tukey's HSD was performed *post-hoc* to determine significant differences between individual groups of interest while accounting for multiple comparisons. Differences were considered significant for *p* < 0.05. A minimum sample size of 7 was used for each group, corresponding to a statistical power of 0.91 and effect size capable of being detected of 0.96.

## Results

### L4-GFP transfection and ribosomal expression in Schwann cells

**Figure 2:**
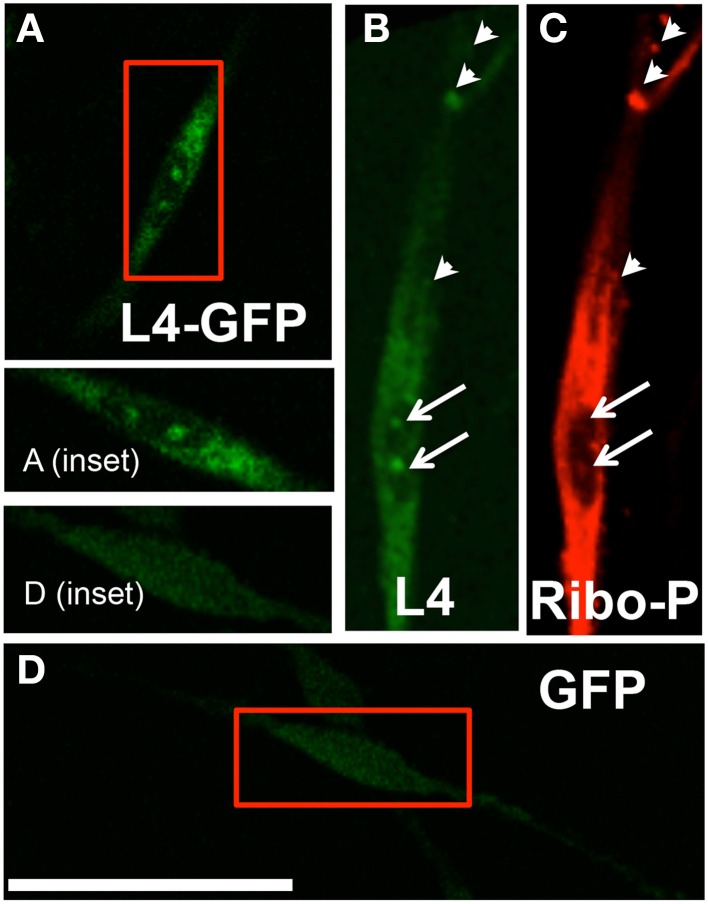
**(A)** Typical pattern of a transfected Schwann cell expressing L4-GFP (magnified in inset); **(B)** A separate schwann cell immunostained with anti-L4; **(C)** Schwann cell immunostained with anti-Ribo-P shows co-localization with L4 expression, based on Manders co-localization analysis (arrows indicate nucleolar labeling; arrowheads indicate prominent co-localizing puncta); **(D)** Transfected Schwann cell expressing GFP (magnified in inset) shows continuous low-level expression throughout cell, including the nuclear compartment, and differs from the L4-GFP labeling pattern; Scale bar: 50 μm.

We confirmed and characterized transfection of SCs with the L4-GFP plasmid (Figure 2A). Only ~15% of cells were successfully transfected; however, comparison of localization patterns of L4-GFP in transfected cells with localization patterns observed in cells immuno-labeled for ribosomal protein L4 showed strong agreement (Figure 2B). Of particular note were the characteristic punctate expression within the nucleolus, which has been observed previously for L4-GFP as well as several other ribosomal subunits (Krüger et al., 2007), and higher relative fluorescence within the cell body compared to the projection of the SC. Immunolabeled L4 also co-localized with Ribo-P, a marker for phosphorylated ribosomes, both inside and outside of the nucleus (Figure 2C; Nucleus: Manders M1 = 0.61; M2 = 0.79; Extranuclear: M1 = 0.58; M2 = 0.89). The expression profile of L4-GFP also differed greatly from that of GFP expression (Figure 2D), which was evenly distributed in low levels throughout the entirety of the SC.

### Ribosomal expression characteristics within Schwann cell projections

Following successful transfection, SCs associated with neurons were identified based on the position of cell bodies and projections of each cell type. We investigated these cells to determine the development of ribosomal distributions within SC projections over time and following myelination induction (Figure 1A). We compared early (Days 1 and 3) and late (Day 7 No AA) time points for untreated cells, and for the late time point, also cells treated with ascorbic acid at day 4 to induce myelination (Day 7AA).

**Figure 3:**
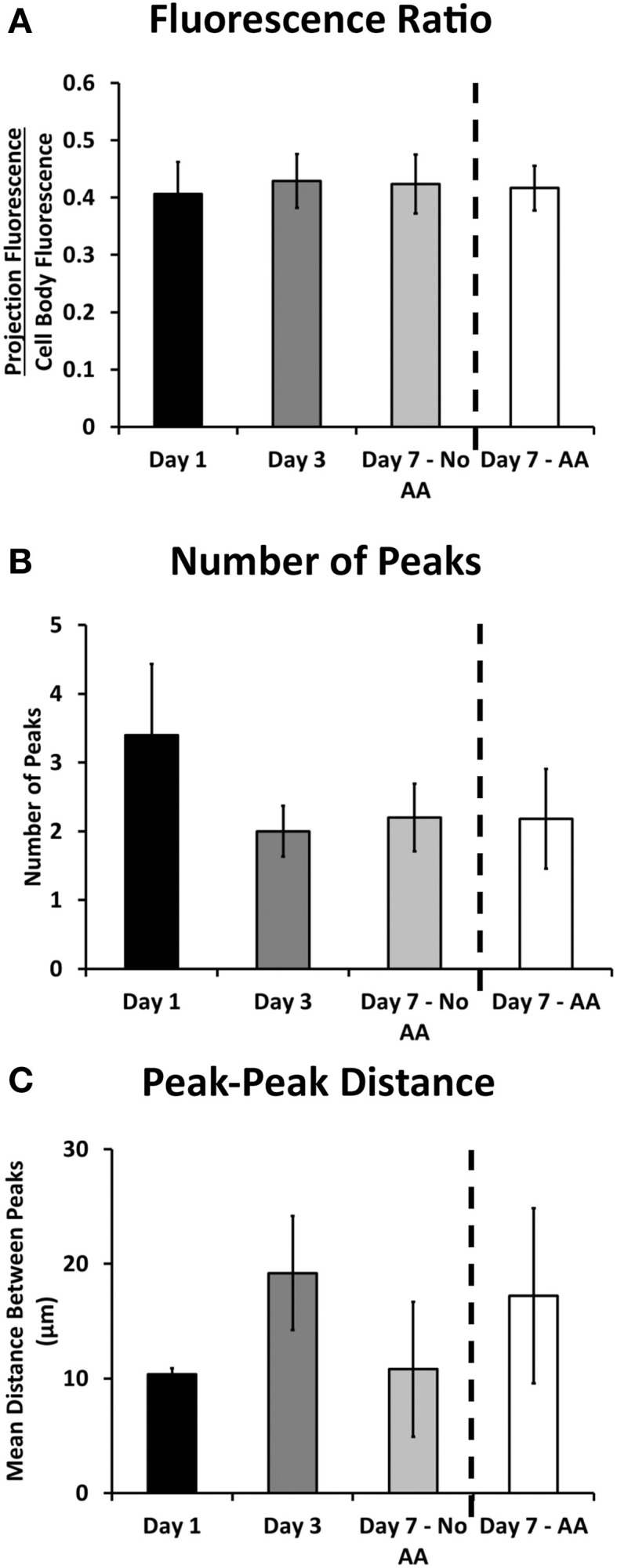
**Ribosomal expression in the projections of Schwann cells remained relatively consistent throughout the experimental proceedings according to a number of metrics. (A)** The ratio of fluorescence in the projection compared to the cell body remained consistent throughout the experimental groups; **(B)** The number of observed ribosomal peaks (or puncta) did not vary significantly throughout the experimental groups; **(C)** The distance between peaks varied albeit not significantly throughout the experiment (Means ± SEM). *Statistics: 1-way ANOVA*.

The ratio of cell body fluorescence to projection fluorescence (average cell body pixel value/average projection pixel value) was used to determine any difference in bulk localization of ribosomes. This ratio was calculated for sub-saturation fluorescence, and was independent of any cell to cell differences in fluorescence expression. The ratios at each time point were consistent, falling between 0.406 and 0.429 (*p* = 0.486; One-Way ANOVA, Figure 3A), suggesting no net increase or decrease in projection expression levels among all experimental groups.

Bright, stationary ribosomal patches dotting the Schwann cellular projection were consistently observed at each time point. The peaks were characterized by a minimum 3x increase in fluorescence over the average projection expression level. The number of these peaks did not change amongst the experimental groups (between 2.2 and 3.4; *p* = 0.77; One-Way ANOVA, Figure 3B). Additionally the peak to peak distance did not vary (*p* = 0.605 One-Way ANOVA, Figure 3C). Several additional morphological and fluorescence parameters of interest, including projection length, total fluorescence, and distances of peaks from cell bodies were also calculated (Table 4). These also yielded no significant differences. This evidence points to an early establishment and inherent stability of the ribosomal clusters following SC alignment with neurons, independent of time or early myelination.

**Table 4:** **Projection data**.

	**Day 1**	**Day 3**	**Day 7—No AA**	**Day 7 AA**	***P*-value (ANOVA)**
Projection length (μm)	62.33±9.08	80.19±10.423	71.58±9.52	93.15±24.45	0.486
Cell body Avg. intensity	12.94±2.4	12.65±2.1491	13.28±1.58	13.89±1.29	0.496
Projection Avg. intensity	4.68±0.46	4.63±0.69534	5.22±0.41	5.63±0.72	0.77
Projection/Cell body ratio	0.41±0.06	0.43±0.046995	0.42±0.05	0.42±0.04	0.605
Number of peaks	3.4±1.03	2±0.36927	2.2±0.49	2.18±0.72	0.663
Avg. distance peak to cell body (μm)	24.78±6.28	35.04±10.52	30.75±12.83	41.62±17.79	0.525
Avg. distance peak to peak (μm)	10.38±0.51	19.19±4.9773	10.8±5.88	17.22±7.65	0.713

*Additional values from projection analysis show little variability between experimental groups, p-values based on 1-ANOVA of experimental group*.

### Cytoskeletal characteristics of ribosomal clusters in Schwann cells

**Figure 4:**
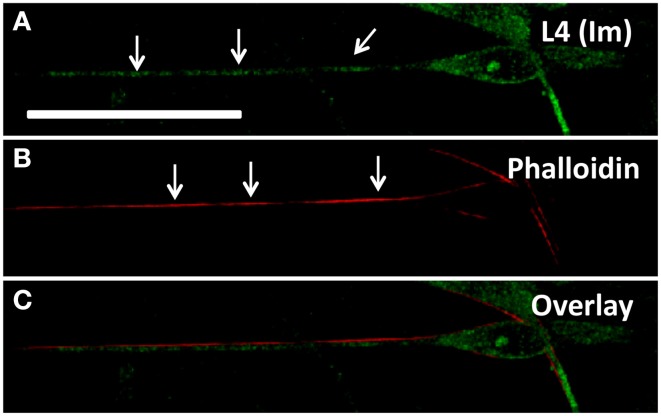
**(A)** Schwann cell immunostained for L4 shows expression throughout the projection with specifically punctate regions (arrows) corresponding to ribosomal rich regions; **(B)** Phalloidin staining highlights cortical regions rich in actin (arrows) but not necessarily corresponding with ribosomal rich regions **(C)**. Scale bar: 50 μm.

Based on observations of apparently stable ribosomal clusters, we tested whether any particular cytoskeletal components were associated with local ribosomal clusters. In neurons, previous reports indicated high densities of F-actin subjacent to ribosome-enriched periaxoplamic ribosomal plaques (PARPs) (Koenig and Martin, 1996). We therefore tested whether a similar phenomenon also occurred in SCs, by examining co-localization of immunolabeled L4 and phalloidin-labeled F-actin. We did observe bright regions of actin along the SC projection, interspersed with dim regions, enabling analysis of co-localization with ribosomal clusters. However, L4 clusters in SC projections showed no specific co-localization or apparent proximity to F-actin in SCs alone (Manders = 0.179 ± 0.082), in the presence of neurons without induction of myelination (Day 1; Manders = 0.341 ± 0.288) and in the presence of neurons following induction of myelination (Day 7AA; Manders = 0.197 ± 0.113) (Figures 4A–C). The apparent offset of phalloidin and L4 is likely resultant from cortical localization of actin and central cytoplasmic localization of L4. We also examined localization of ribosomes relative to microtubules, based on co-localization of Ribo-P with tubulin. However, fluorescent intensity of tubulin was uniformly consistent along the projection and thus precluded quantitative analysis of co-localization (data not shown).

### Ribosomal transport characteristics within Schwann cell projections

**Figure 5:**
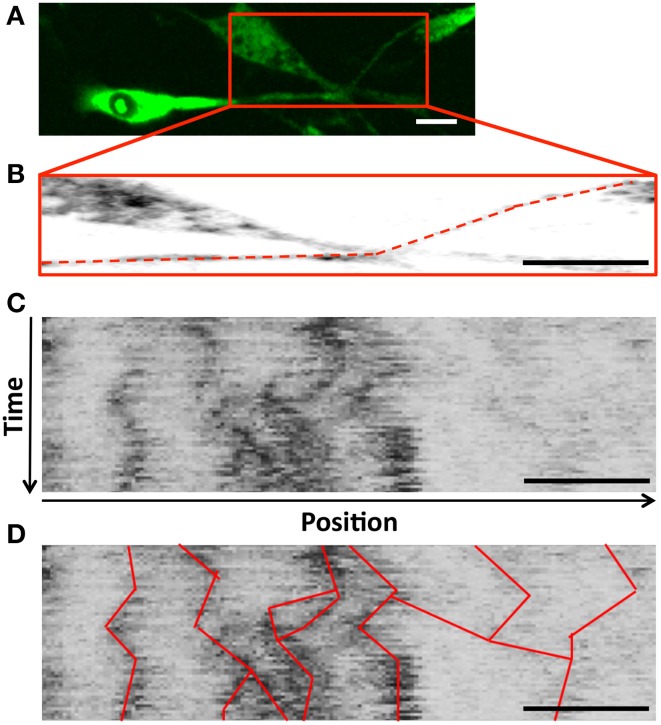
**(A)** Schwann cell projections were cropped and traced to develop **(B)** Sample trace (dashed line) used to develop a kymograph **(C)** a kymograph (y-axis: time, increasing downward; x-axis: position along the projection, with anterograde to the right); **(D)** Trajectories for puncta observed in consecutive frames were traced on the kymograph to identify ribosomal movement throughout the projection, and allow for quantification of transport parameters during the course of the experiment; Scale bar: 20 μm, duration 5 min.

Though densities were apparently stable, movies of L4-GFP dynamics, captured using time-lapse fluorescence microscopy, revealed movement of less intense L4-GFP puncta between densities. Thus, to examine whether ribosomal transport contributed to the maintenance of densities, we next quantitatively characterized the movement of L4-GFP using kymograph analysis, which allowed the observation of moving L4-GFP particles, and the characterization of puncta directionality, velocity, and duration of movement (Figure 5). Individual puncta were treated as single particles, independent of fluorescence intensity.

**Figure 6:**
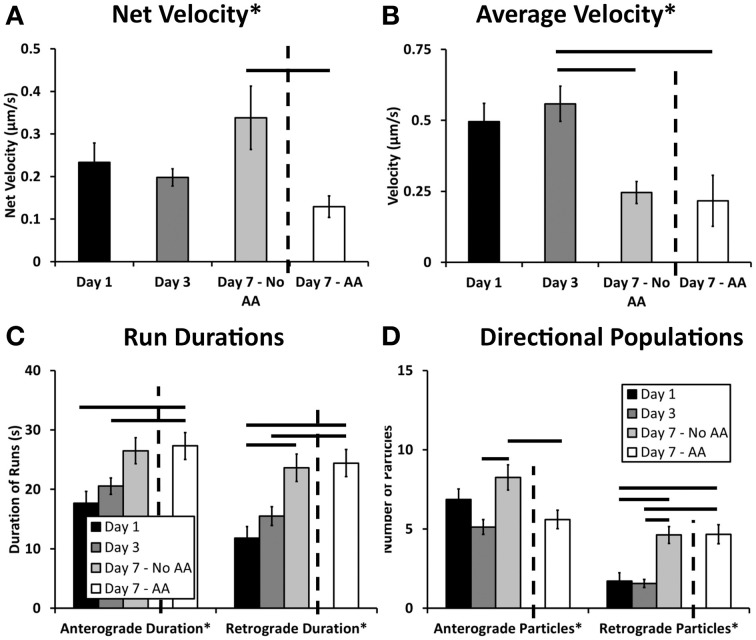
**Kymograph analysis provided a clear picture of the dynamics of ribosomal transport**. **(A)** Net velocity, a measurement that provides a relative comparison of the bulk transport occurring in the cell, showed increased levels after 7 days. These levels were decreased when the cultures were treated with ascorbic acid indicating an effect of this treatment and early myelination on ribosomal transport; **(B)** In both day 7 treatment groups, the average velocity was decreased compared to day 3; **(C)** Anterograde and retrograde durations varied over the course of the experiment showing significant differences between early and late time points; **(D)** The number of anterograde particles increased after 7 days in the absence of ascorbic acid compared to both the day 3 and day 7 ascorbic acid treated groups. Additionally, there was an increase in retrograde particles in both the day 7 groups compared to the earlier time points, likely influencing the observed changes in net velocity (Means ± SEM) *Statistics: 1-way ANOVA* (^*^ *p* < *0.05); Tukey HSD* (**−** *p* < 0.05).

The summation of net velocity for each individual L4-GFP particle within a given movie offers a summary of directionality and extent of movement within a cell during our imaging period. Normalization of this displacement to the imaging duration yields net velocity, which enables comparison with bulk transport rates captured over a longer time frame (Love et al., 2012; Pathak et al., 2013). There are significant differences in net velocity of ribosomes over the course of Schwann cell development (*p* = 0.0038, One-Way ANOVA, Figure 6A). In particular, though ribosomal transport is anterograde (away from the Schwann cell body) at all-time points, ribosomal transport at Day 7 increases in the absence of ascorbic acid, but is suppressed in the presence of ascorbic acid. (Mean ± SEM; No AA: 0.388 ± 0.074; AA: 0.129 ± 0.025, *p* = 0.00189, Tukey's HSD).

We further examined individual transport parameters to determine their contribution to net velocity in each experimental group. The comprehensive data set, including statistical analysis, is summarized in Table 5. For clarity, we discuss key findings below.

**Table 5:** **Transport data**.

	**Day 1**	**Day 3**	**Day 7—No AA**	**Day 7 AA**	***P*-Value(ANOVA)**
Net velocity (μm/s)	0.233±0.046	0.198±0.02	0.338±0.074	0.129±0.025^†^	0.0038
Avg. velocity (μm/s)	0.496±0.064	0.558±0.062	0.246±0.039^∧^	0.216±0.09	0.00258
Avg. Ant. Vel. (μm/s)	0.765±0.055	0.802±0.065	0.624±0.086	0.621±0.075	0.178
Avg. Ret. Vel. (μm/s)	−0.52±0.234	−0.38±0.122	−0.525±0.148	−0.474±0.08	0.848
Tot. Ant. Part	6.86±0.67	5.13±0.46	8.25±0.8^∧^	5.6±0.58^†^	0.00628
Tot. Ret. Part.	1.71±0.52	1.56±0.26	4.63±0.53^*^^∧^	4.67±0.61	0.00000615
Ant. Duration (s)	17.65±2.01	20.55±1.37	26.49±2.18^*^	27.32±2.21^*^^∧^	0.00525
Ret. Duration (s)	11.79±1.95	15.51±1.58	23.62±2.3	24.41±2.28^*^^∧^	0.000326

Values from transport analysis showing variability among velocities and particle distributions between groups, p-values passed on 1-ANOVA (Tukey HSD, p < 0.05 compared to

* *Day 1*,

∧ *Day 3*,

† Day 7—No AA)

The average ribosomal velocity was also dependent upon the developmental state of the Schwann cell (*p* = 0.0025 One-Way ANOVA, Figure 6B). Most notably, there was a significant reduction in average velocity from Day 3 (0.56 μm/s) to Day 7 (No AA: 0.25 μm/s; AA: 0.22 μm/s) irrespective of ascorbic acid treatment (Day 3–Day 7 No AA: *p* = 0.041; Day 3–Day 7 AA: *p* = 0.004, Tukey's HSD). While the reduction in average velocity might be expected in the case of Day 7AA, where net velocity is also reduced, this was unexpected in the case of the untreated Day 7 group, where net velocity in fact increases. This apparent paradox indicates that other phenomena are integral in determining the net velocity.

Average particle run durations varied between 18 and 27 s in the anterograde direction and 12 and 24 s in the retrograde direction (Figure 6C). There was a trend toward increasing durations in both the anterograde and retrograde directions. Significance differences were observed in both directions between early time points and Day 7AA (Anterograde: Day 1–Day 7 AA: *p* = 0.016; Day 3–Day 7 AA: *p* = 0.038; Retrograde: Day 1–Day 7AA: *p* = 0.0018; Day 1–Day 7 AA: 0.0070). Additionally a difference was observed between Day 7 in the absence of ascorbic acid and Day 1 in the retrograde direction (*p* = 0.011).

A final component of net velocity is the number and proportion of particles being transported in each direction. There were significant differences in both particle populations (Anterograde: *p* = 0.006; Retrograde: *p* = 0.000006, One-Way ANOVA, Figure 6D). Of note in the case of the anterograde particles is the significant increase in anterograde particles from Day 3 (5.125 particles) to Day 7 (No AA: 8.25 particles) in the absence of ascorbic acid (*p* = 0.005, Tukey's HSD), but not the presence of ascorbic acid (AA: 5.6 particles; *p* = 0.918, Tukey's HSD). This increase in particle number thus may be a key factor contributing to the observed increase in net velocity.

### Multiple regression

We performed multiple regression analysis on a particle by particle basis to determine the individual contributions of each component measurement (independent variables) to the resultant net velocity (dependent variable). A complete summary of regression results is found in Table 6. At early time points, anterograde velocity had a strong influence on net velocity (Day 1: 0.431; Day 3: 0.474), with additional contributions from directional durations (Day 1 total duration: 0.537; Day 1 retrograde duration: -0.595; Day 3 anterograde duration: 0.474). At later stages, irrespective of ascorbic acid treatment, directional (anterograde) duration continued to contribute most strongly to net velocity (Day 7 AA: 0.630; Day 7 no AA: 0.925). Though the effect of anterograde velocity was sharply diminished, average velocity also contributed strongly to the model (AA: 0.525; No AA: 0.480). Thus, under the assumption that each parameter is modulated, at least partially, by a different set of biological influences, these results suggest that regulation of ribosomal transport in Schwann cells evolves temporally, but is not influenced by early myelination.

**Table 6:** **Multiple regression**.

	**Day 1**	**Day 3**	**Day 7 No AA**	**Day 7 AA**
Avg. Vel.	0.021	0.022	0.480	0.525
Tot. Time	0.537	0.003	−0.352	−0.250
Avg. Ant. Vel.	0.413	0.474	−0.041	−0.041
Time Ant.	0.000	0.474	0.952	0.630
Avg Ret. Vel.	0.028	0.042	−0.060	−0.081
Time Ret.	−0.595	−0.244	0.295	−0.028

*β-weights for multiple regression of z-scored data for independent variables regressed against the dependent variable net velocity show a shift from anterograde velocity dependence at early time points (Days 1 and 3) to anterograde time dependence at later time points (Day 7)*.

### Ribosomal transport model

**Figure 7:**
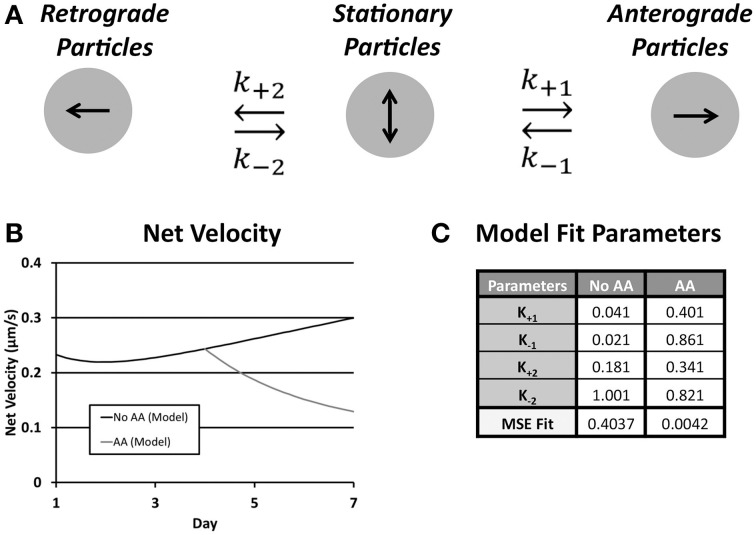
**(A)** Schematic of the rate kinetic model shows possible particle states and transition rate constants between the three states; **(B)** Plot of net velocity from optimized model output; **(C)** Following ascorbic acid treatment the rate of conversion from anterograde to stationary (k_−1_) as did the conversion rate from stationary to both anterograde (k_+1_) and retrograde (k_+2_) increased resulting in an overall reduction in net velocity. MSE fit agreement was strong in both treatment groups.

A limitation of the narrow temporal window during which transport was assessed as well as a finer understanding of factors underlying outcomes from multiple regression analysis was an inability to examine directional transitions in particle movement. Thus, we developed a simple yet informative kinetic model, driven and validated by our experimental data, which was captured over a time frame of minutes, on net transport, which was measured over a time frame of days (Figure 7). The model determined a set of rate constants that best fit experimental parameters for net velocity and the number of anterograde and retrograde particles, based on computed mean squared error (Table 3). Numerical solution of the system of differential equations was stable. Model fits were extremely strong (No AA: MSE = 0.4037; AA: MSE = 0.0042), and were unique within the wide range of allowed parameter values. Sensitivity analysis was also performed for each rate constant over this range of values (Supplemental Figure 1), and revealed that the model was most sensitive to changes in k_+1_ followed by k_+2_.

The transition of rate constants upon addition of ascorbic acid provided information about the shift in ribosomal transport during onset of myelination. In both cases the rate of conversion from stationary to anterograde particles increased (k_+1_, No AA: 0.041; AA: 0.181). This was accompanied by an even greater increase in the rate of conversion of anterograde particles to stationary (k_−1_, No AA: 0.021; AA: 0.861). This caused an inversion of the ratio of the rate constants shifting the preference of particles in this state toward the stationary population. Similarly, there was an increase in the rate of particles converting from stationary to retrograde (k_+2_, No AA: 0.181; AA: 0.341). Combined with the decrease in the rate of conversion form stationary (k_−2_, No AA: 1.001; AA: 0.821) increased the favorability of the retrograde position following ascorbic acid treatment. This led to a decrease in anterograde particles and an increase in retrograde particles resulting in a decreased net velocity observed within the model. Cumulatively, these results suggest that the increases in k_−1_ and k_+2_ are the likely drivers of decreased anterograde and increased retrograde particle number, as well as, decreased net velocity following induction of myelination.

## Discussion

The localization, dynamics, and activity of protein synthetic machinery have received increased attention in neurons (Gould and Mattingly, 1990; DiStefano et al., 1992; Morris and Hollenbeck, 1995; Twiss and van Minnen, 2006; Pathak et al., 2013), motivated in large part by the demands imposed by neuronal polarity and geometry. Local protein synthesis has been less extensively studied in Schwann cells, though they also extend long projections, and thus also face similar metabolic, structural, and biochemical demands (Jacobs and Cavanagh, 1969; Colman et al., 1982; Gould and Mattingly, 1990). Additionally, recent evidence has suggested that Schwann cells are a source of mRNA and ribosomes for neurons recovering from injury, providing additional rationale for localizing protein synthetic machinery to SC projections (Court et al., 2008, 2011).

This study used a combined experimental and theoretical approach to examine the localization and movement of ribosomes within the projections of Schwann cells at high resolution. While several important studies have documented the presence of mRNA and ribosomes in SC projections (Colman et al., 1982; Trapp et al., 1987, 1988; Griffiths et al., 1989; Gould and Mattingly, 1990), the development and plasticity of ribosomal populations locally remain unknown. Our overall hypothesis was that local populations would be developed early during projection extension to enable projection outgrowth. However, such populations would then require supplementation, both for maintenance, given the ~40 h half-life of ribosomes (Weber, 1972), as well as to sustain increased interactions with neurons, which may be stably myelinated for years (Elson et al., 2004). Our results support our original hypothesis of anterograde transport of ribosomes developing ribosomal populations in the projections of Schwann cells during initial projection elongation. In early phases of myelination, ribosome trafficking decreases slightly as expected, in line with a shift toward a maintenance regime.

### Stable ribosomal populations

Localization patterns of L4-GFP in projections of transfected Schwann cells showed strong agreement with the localization patterns of immunolabeled L4. Two characteristics, seen both in transfected cells and immuno-labeled cells, are of particular note. First, similar to MBP mRNA distributions described previously (Trapp et al., 1987, 1988; Gould and Mattingly, 1990), there was a level of punctate ribosomal expression throughout the projection. It is possible that these ribosomes are associated with RNA granules of varying size and function, as suggested for oligodendrocytes (Thomas et al., 2005). Second, there were 2–3 stable peaks of high fluorescence intensity within each Schwann cell projection, likely pointing to locations of ribosomal clustering. These peaks were apparent upon initial projection extension on day 1 and persist throughout the 7 days of observation both in the presence and absence of ascorbic acid. This pattern too is similar to MBP mRNA densities within SC projections (Gould and Mattingly, 1990). Future studies will reveal whether ribosomal densities synthesize proteins for stages of myelination or participate in ribosomal transfer to neurons (Gould and Mattingly, 1990; Court et al., 2008) at structures yet to be formed. For instance, Schmidt-Lanterman incisures have been suggested as the region of ribosomal transfer following injury and occur at similarly spaced intervals (~25–30 μm) (Buchthal et al., 1987) as the observed ribosomal clusters (Buchthal et al., 1987; Court et al., 2008). Interestingly, while protein synthesis does not appear to extend into the Schmidt-Lanterman incisures there does seem to be significant ribosomal localization to the surface network in the region adjacent the incisures (Gould and Mattingly, 1990).

To probe a possible structural basis for observed ribosomal densities, cells were co-labeled for RPL4 and cytoskeletal components. In neurons, ribosomal clusters (periaxoplasmic ribosomal plaques) are observed within axons and cluster around regions enriched in F-actin (Koenig and Martin, 1996). Similarly, Schmidt-Lanterman incisures are enriched in F-actin (Trapp et al., 1989; Court et al., 2008, 2011) However, co-labeling of ribosomal subunits with F-actin and tubulin failed to display any obvious co-localization with ribosomal clusters (Figure 4). On the other hand, it is possible that a specific set of actin- or tubulin-associated proteins rather than the filaments themselves may play a role in docking or sequestering ribosomes (Wilhelm and Vale, 1993).

### Ribosomal movement

By Day 1 after neuronal contact, newly synthesized ribosomes containing L4-GFP were already distributed throughout Schwann cell projections. Additionally, distributions revealed minimal changes between Day 1 and Day 7, in the presence or absence of ascorbic acid. Though it was somewhat surprising that ribosomal densities were stabilized so rapidly, also of interest was the activity of more dynamic populations of directionally moving ribosomes observed using time-lapse microscopy. Kymograph analysis, which captured ribosomal transport characteristics at each experimental time point at high resolution, enabled us to quantify and compare transport parameters in greater detail.

Individual transport parameters were integrated into an estimate of net movement (net velocity) of ribosomal populations, which served as a rough surrogate for ribosomal demand at a given stage of growth or myelination. The nucleus was the source of newly synthesized fluorescent ribosomal subunits, and as such explains the net anterograde velocities for each experimental group. Bulk rates were ~0.1–0.3 μm/s (~8–25 mm/day), which corresponds to an intermediate rate of transport in a neuron (Brown, 2000). Interestingly, at early time points, the net velocity remained quite stable, but diverged at Day 7, dependent upon ascorbic acid treatment; ascorbic acid treatment led to an overall reduction in net anterograde velocity, whereas the absence of ascorbic acid led to an increase.

The cellular processes driving reduced net velocity in the presence of ascorbic acid are mostly unexplored. Reduced ribosomal demand may simply represent a more focused diversion of translational machinery to the myelination process (Lemke and Chao, 1988). On the other hand, several Schwann cell activities appear to be coupled to early stages of SC-neuron contact and myelination. Structurally, neuronal contact alters the nucleation, polarity, and distribution of microtubules within myelinating Schwann cells (Kidd et al., 1994). In addition, consistent with reduced cellular extension and crawling during neuronal interactions, mobility of Schwann cell membranes and their associated cytoskeleton is reduced and adhesion is enhanced (Feltri et al., 1994; Nobes and Hall, 1999; Love et al., 2012). Alternatively, cell morphological changes may reflect a redistribution or change in the stability of microtubules, which may result from Schwann cell differentiation and maturation. It is not inconceivable that such structural changes during early stages of Schwann cell differentiation and myelination could influence the directionality, duration, and overall levels of ribosomal transport. Indeed, initiation of myelination in regenerating nerves results in altered expression and distribution of several genes and proteins in Schwann cells, including neurofilaments (Fabrizi et al., 1997), TGF-beta (Scherer et al., 1993), P0 glycoprotein (LeBlanc et al., 1992), and ion channels (Chiu et al., 1994).

While there are drastic changes in net velocity with the initiation of myelination, the average anterograde and retrograde velocities did not change, suggesting that the regulation of molecular motor activity is changed, not the responsible motors themselves. While it is unclear which motors are responsible for ribosomal transport in Schwann cells, in other cellular systems the distribution of ribosomes depends on early endosome trafficking of both kinesin-3 and dynein (Higuchi et al., 2014; Palacios, 2014). Parameters that might change include binding affinities, number of available motor proteins, and initiation of transport all of which would affect net velocity but not the individual components of velocity (Lipowsky et al., 2010). The observed decrease in net ribosomal trafficking is attributable to increased levels of retrograde moving particles as well as a reduction in anterograde particles. The increase in retrograde particles at Day 7, for both ascorbic acid-treated and untreated cells, are perhaps due to ribosomal recycling (Lafontaine, 2010). The reduction in anterograde transport may reflect increased demand for protein synthesis, which we speculate occurs in stationary ribosomes docked on structures such as those discussed above. Despite this reduction in anterograde movement, the fact that the net velocity remains positive at these times suggests that ribosomes are required in the periphery on an ongoing basis, perhaps to maintain and replenish the stable ribosomal pools.

### Theoretical modeling of ribosomal transport

To further understand differences in transport, we used our high-resolution assessment of transport to develop and validate a simple, yet powerful, data-driven rate kinetic model of ribosomal transport. This model was particularly effective at describing the transfer of particles from one movement state (stationary, anterograde, or retrograde) to another, providing insight into underlying transport dynamics not amenable to other types of analyses.

For example, population analysis indicated that both anterograde and retrograde populations decreased, and as a consequence, the magnitude of net velocity was also reduced. Our model enabled us to assess whether these reductions in anterograde and retrograde populations resulted from increased transition from moving to stationary particles, decreased transition from stationary to moving particles, or both. For our case, the model revealed an increased rate of conversion from anterograde to stationary (higher k_−1_), an increased rate of conversion from stationary to retrograde (higher k_+2_), and a slightly lower rate of conversion from retrograde to stationary (lower k_−2_) upon addition of ascorbic acid. These three changes thus drove the observed reduction in anterograde movement and net velocity.

The model also raises hypotheses about different mechanisms for the decrease in both anterograde and retrograde populations. The increased conversion of anterograde particles to stationary may be resultant of myelin compaction and other restrictions on transport within the projection compartment. The increased recruitment of retrograde particles may result from a decreased demand for local ribosomes due to their very specialized functions in the myelination process. Such possibilities may be considered and tested in future experiments.

Despite the unique solution provided by the model the simplicity of the model possesses some limitations based on experimental observations and procedures. This includes the lack of time points for the model fit for ascorbic acid due to the differential fit of these cultures at earlier time points when ascorbic acid was lacking. Additionally, omitted from the model was the ability of particles to transfer directly from anterograde to retrograde. This was done because in the time scale of the imaging (5 s) we did not observe any particles that directly transitioned. Rather they would become stationary for a short period of at least 15 s before transitioning between states. Finally, it is worth comparing our modeling approach to a number of other excellent theoretical models of intracellular transport. Such models differ in a variety of features, including the identity of cargoes modeled (e.g., a single generic cargo vs. populations of specific cargoes), motor configurations on a given cargo, details on mechanisms by which cargoes move, stop, or change their direction, and the modeling strategy itself (Badoual et al., 2002; O'Toole et al., 2008; Lipowsky et al., 2010; Bressloff and Newby, 2013; Gumy et al., 2014). These models also span a variety of temporal and spatial scales. Our model, based on the original rate kinetics framework of Smith and Simmons (Smith and Simmons, 2001; Kuznetsov, 2010; Zadeh and Shah, 2010), exploited experimental measurements in single cargoes to answer questions related to the transition of directional bulk cargo populations. In this context, there were minimal effects of spatial gradients and diffusion on ribosomal distribution within our time scale of interest. Experimentally, visible fluorescent puncta did not move in a manner consistent with diffusion [i.e., runs were directional for >15 s, and thus attributed to motor-driven movement (convection)]. Also, background fluorescence, likely representing soluble ribosomal subunits, remained evenly distributed through the imaged regions of the axon during imaging. Thus, in light of the absence of any experimental evidence, diffusion did not contribute appreciably to the model, and was ignored. Similarly, no gradients in any transport parameter were detectable along the length of the projection (possibly due to the low number of moving particles along each projection), and thus were also not factored into our final model.

## Conclusions and future directions

This study provides a first quantitative look into the establishment of ribosomal populations within Schwann cells following neuronal contact and myelination, and transport changes associated with a hypothesized change in demand for a local protein synthesis source. While others have shown that proteins are synthesized locally in Schwann cell projections and are required for myelination, we are the first to observe, quantify, and model the process dynamics during early development and myelination. The observed ribosomal transport decrease into the Schwann cell projection is likely tied to a decrease in demand for protein synthesis. However, many questions remain to be answered about how and why such dynamics occur, including which motor proteins are at work, the effect of early and late myelination on local protein synthesis, and the role, if any, early ribosomal localization may have on the proposed process of transcytosis(Court et al., 2008). Our initial findings open the possibility for a number of future studies that might be integral in developing translational clinical solutions for nervous system disease and injury.

### Conflict of interest statement

The authors declare that the research was conducted in the absence of any commercial or financial relationships that could be construed as a potential conflict of interest.

## Supplementary material

Supplemental Figure 1 **Model traces for varying rate constants show degree of sensitivity for the unique solution**. No AA groups were highly sensitive to forward rate constants, k_+1_ **(A)** and k_+2_ **(C)**, yet slightly less sensitive to variations in reverse rate constants, k_−1_ **(B)** and k_−2_ **(D)**. Similar results held for parameter variations in the AA treated model with higher sensitivity to changes in both forward rate constants, k_+1_ **(E)** and k_+2_ **(G)**, and lower sensitivity to the changes in the reverse rates, k_−1_ **(F)** and k_−2_ **(H)**.
