# Supplementary figures and images for: Ribosomal trafficking is reduced in Schwann cells following induction of myelination
Source: Front Cell Neurosci. 2015 Aug 19;9:306. doi: 10.3389/fncel.2015.00306 (PMC4541260; doi:10.3389/fncel.2015.00306)

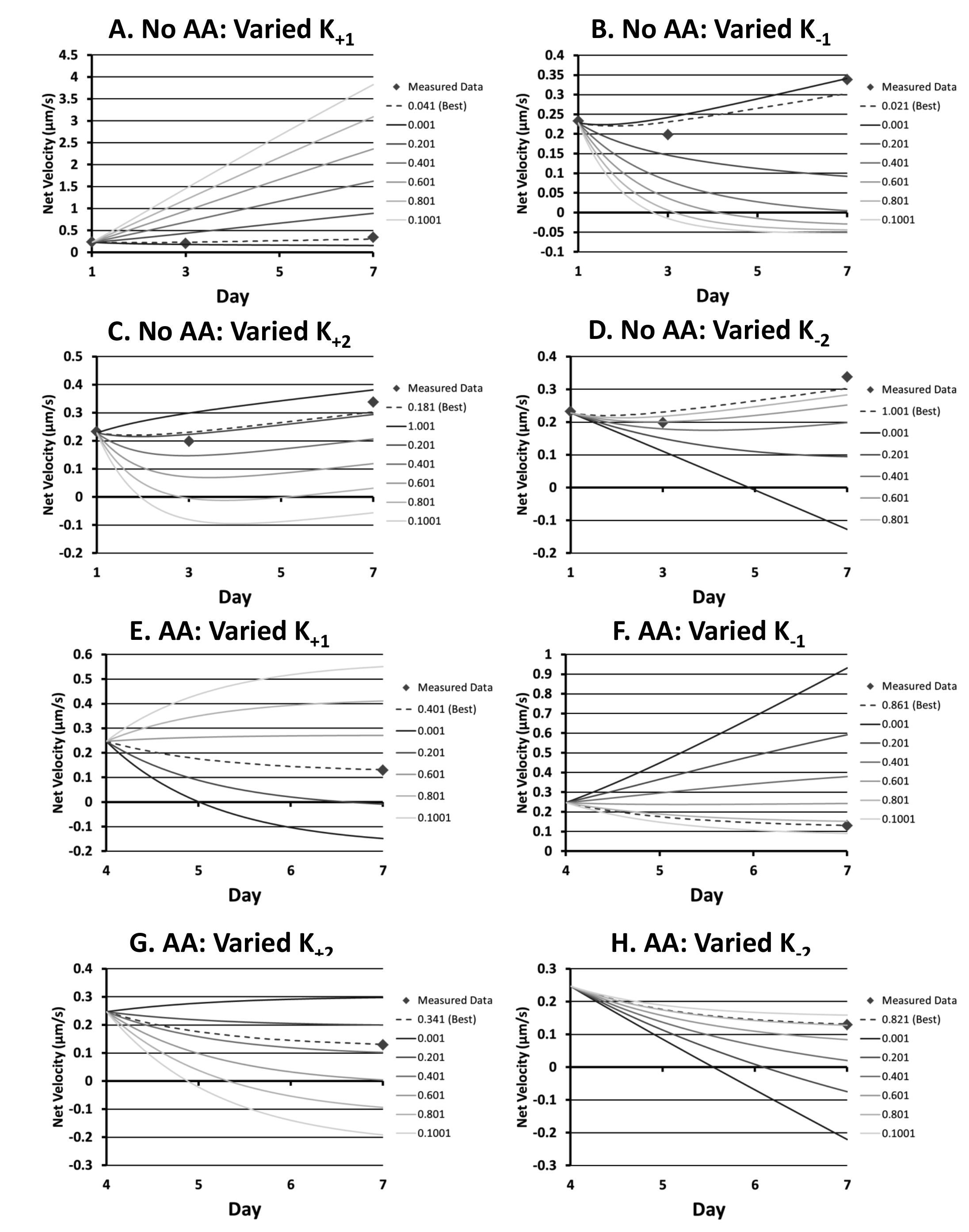

Supplement: Supplemental Figure 1 — Model traces for varying rate constants show degree of sensitivity for the unique solution. No AA groups were highly sensitive to forward rate constants, k+1 (A) and k+2 (C), yet slightly less sensitive to variations in reverse rate constants, k−1 (B) and k−2 (D). Similar results held for parameter variations in the AA treated model with higher sensitivity to changes in both forward rate constants, k+1 (E) and k+2 (G), and lower sensitivity to the changes in the reverse rates, k−1 (F) and k−2 (H). [file Image1.TIF]
